# The effect of surface electromyography biofeedback on the activity of extensor and dorsiflexor muscles in elderly adults: a randomized trial

**DOI:** 10.1038/s41598-019-49720-x

**Published:** 2019-09-11

**Authors:** Ana Belén Gámez, Juan José Hernandez Morante, José Luis Martínez Gil, Francisco Esparza, Carlos Manuel Martínez

**Affiliations:** 1 Physiotherapy Service, “Sagrado Corazón” Hospital, Malaga, Spain; 2 Faculty of Health and Life Sciences, Catholic University of Murcia, Murcia, Spain; 3 Physiotherapy Service, Arrixaca Hospital, Murcia, Spain; 4 International Chair of Cineanthropometry, Catholic University of Murcia, Murcia, Spain; 5 IMIB - Experimental Pathology Service, Arrixaca Hospital, Murcia, Spain

**Keywords:** Geriatrics, Geriatrics, Brain injuries, Brain injuries

## Abstract

Surface electromyography-biofeedback (sEMG-B) is a technique employed for the rehabilitation of patients with neurological pathologies, such as stroke-derived hemiplegia; however, little is known about its effectiveness in the rehabilitation of the extension and flexion of several muscular groups in elderly patients after a stroke. Therefore, this research was focused on determining the effectiveness of sEMG-B in the muscles responsible for the extension of the hand and the dorsiflexion of the foot in post-stroke elderly subjects. Forty subjects with stroke-derived hemiplegia were randomly divided into intervention or control groups. The intervention consisted of 12 sEMG-B sessions. The control group underwent 12 weeks (24 sessions) of conventional physiotherapy. Muscle activity test and functionality (Barthel index) were determined. Attending to the results obtained, the intervention group showed a higher increase in the average EMG activity of the extensor muscle of the hand and in the dorsal flexion of the foot than the control group (*p* < 0.001 in both cases), which was associated with an increase in the patients’ Barthel index score (*p* = 0.006); In addition, Fugl-Meyer test revealed higher effectiveness in the lower limb (*p* = 0.007). Thus, the sEMG-B seems to be more effective than conventional physiotherapy, and the use of this technology may be essential for improving muscular disorders in elderly patients with physical disabilities resulting from a stroke.

## Introduction

One of the greatest medical advancements has been the increase in life expectancy. However, this also means an increase in the prevalence of several age-related diseases, such as stroke^1^. The World Health Organization predicted a dramatic increase in the number of strokes by 2025^2^, and the risk of recurrence after a first stroke has also increased^3^.

Although medical advances have significantly increased the survival rate after a stroke, the main difficulty with this disease is related to the medical aftermath. The restriction of physical functioning due to cerebrovascular damage induced by a stroke (i.e. hemiplegia) is the primary concern related to this disease. In fact, strokes are the most significant cause of long-term disability in the United States^2^, and one of the key public health problems related to this disease is based on the long-term time period before physiological recovery is accomplished. This is an important issue because the failure to recover motor deficits rapidly within a few months after brain damage reduces an individual’s ability to participate in therapy^3^. Thus, the optimisation of the rehabilitation programmes, which must focus on increasing muscle strength and improving the functional condition of the subjects^4^, should be assessed to obtain the best physical reconditioning within the shortest possible time.

At present, rehabilitation therapies include conventional physiotherapy techniques, such as stretching or exercises, but recently their effectiveness has been questioned^5^, and new interventions have been developed. Electrical stimulation is one of the new interventions that has the potential to improve motor activity and performance after a stroke^6^. Although there are various forms of electrical stimulation, surface electromyographic biofeedback (sEMG-B) is becoming increasingly valuable.

A stroke causes neuronal damage and induces a disruption in the voluntary regulation of normal muscle tone, which results in uncontrolled spasticity and muscle weakness in affected patients. It has been suggested that patients might still preserve some unaffected nervous pathways, which are difficult to determine^7^. On the basis of this theory, patients might be able to learn to use these pathways to recover muscle tone regulation. The neurological effects induced by post-stroke rehabilitation treatments need to promote neuroplasticity to improve motor functions^8^, since the recovery of those functions involves re-learning motor skills that are mediated by neuroplasticity^9^, which requires detailed afferent feedback^10^. Studies performed using surface electromyography (sEMG), a non-invasive technique that enables evaluation of the activity of motor units^11^, have reported that there are complex changes in neural and muscular patterns that contribute to muscle weakness after a stroke^12^. Thus, recognition of these patterns should be useful for establishing the appropriate physiotherapy. However, biofeedback is another technique that provides patients with valuable information about the control of their own biological processes, such as muscle activity, thus improving the quality of the rehabilitation^13^.

As a therapeutic option that combines biofeedback and sEMG, sEMG-B is becoming increasingly valuable, because it provides patients with helpful information about their muscle activity by increasing the myoelectric signals and converting these signals into visual and/or auditory signals^7,14–16^. Since the early 1970s, this technique has been used to improve upper extremity, gait, swallowing or upper extremity nerve injuries^11,12^. Enabling a patient to control his/her muscle activity seems to be effective for post-stroke rehabilitation^15,17^.

Nevertheless, the benefits of the use of this technique in the rehabilitation of patients is controversial^4^, and even less is known about its effectiveness in post-stroke hemiplegic elderly patients. This may be due to the limited number of studies performed and the lack of studies concerning the possible benefits for other anatomical regions (such as the lower extremities).

Thus, the present study aimed to investigate the effect of using sEMG-B on the hand extensor muscles and dorsal foot flexor muscles in a cohort of post-stroke hemiplegic elderly patients, and to determine if this technique could improve functional motor activity by increasing the patients’ muscular activity in order to improve their control of the dysfunctional motor patterns established after the stroke. As a secondary objective, changes in the muscle activity of the upper and lower extremities were compared.

## Results

**Figure 1:**
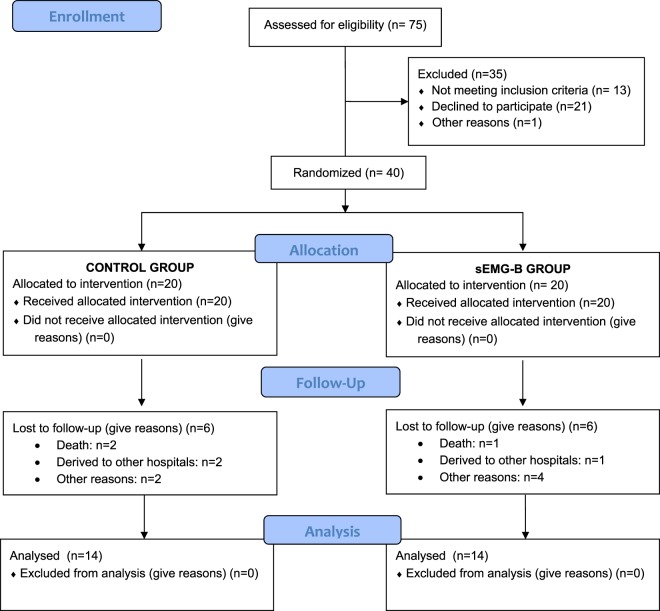
Flow diagram of the trial.

Table 1 shows the general baseline characteristics of all the randomised subjects from both the control group and the sEMG-B group. Of the 40 randomised participants, 28 (control: 14 [50%], sEMG-B: 14 [50%]) completed the trial (Fig. 1). A similar number of subjects withdrew in the control group and the sEMG-B group, so there were no statistical differences in the lost-to-follow-up ratio (*p* > 0.050). Analysis of the effectiveness of sEMG-B was only performed for the participants who completed the intervention (full analysis set).

**Table 1:** Sociodemographic and clinical baseline characteristics of both control and sEMG-B groups.

	CONTROL GROUP (n = 20)	sEMG-B GROUP (n = 20)	*p* (*t*-test,χ^2^)
Sex (women %)	55%	45%	0.525
Age (y)	79 ± 3	78 ± 2	0.144
Stroke story (%)	56%	44%	0.311
Smoking history (%)	48%	52%	0.752
Weight (kg)	70.7 ± 7.8	71.3 ± 8.3	0.792
BMI (kg/m^2^)	26.24 ± 1.95	26.64 ± 1.96	0.519
Barthel Index (score)	56 ± 15	52 ± 18	0.511
Upper limb FUGL- M score	81 ± 10	97 ± 13	0.001
Lower limb FUGL.M score	89 ± 19	154 ± 49	<0.001
Isometric strength (Nw)	19.9 ± 4.3	22.6 ± 10.4	0.734
Kendall score	31 ± 5	38 ± 10	0.085
Daniells grade	3 ± 1	3 ± 1	0.804
Lovett grade	2 ± 1	3 ± 1	0.164
EMG activity of affected upper limb (µV)	32.3 ± 13.2	42.2 ± 9.5	0.011
EMG activity of non-affected upper limb (µV)	60.6 ± 21.4	81.8 ± 35.1	0.030
EMG activity of affected lower limb (µV)	42.2 ± 22.6	40.6 ± 26.6	0.898
EMG activity of non-affected lower limb (µV)	77.0 ± 30.2	77.5 ± 39.8	0.966

Data represent Mean ± SD. BMI: body mass index. FUGL-M: Fugl-Meyer score. EMG activity represents the average EMG activity of a 15-minute session, in µV, analysed with the Neurotrans Myoplus 2 Pro system. Differences between groups were analyzed by Student’s *t*-test.

The demographic and basal clinical characteristic scores of both groups were comparable (Table 1). Only the Fugl-Meyer (FM) score and the upper extremity EMG activity were statistically significantly higher in the sEMG-B group than the control group. However, both groups were similar in terms of age, sex, stroke and smoking antecedents. This similarity was expected, considering the random selection of the subjects in each group.

### The efficacy of sEMG-B on the electromyographic signal activity of affected limbs

**Figure 2:**
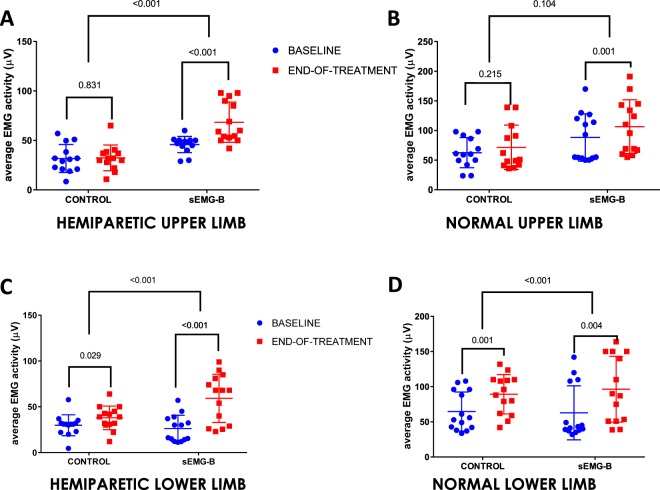
Box-plots with individual activity showing changes in average EMG activity in the hemiparetic and normal extremities. Muscular activity was expressed as the % of the maximum voluntary isometric contraction (%MVIC). Statistically significance values were determined through the ANCOVA analysis. Precise data and statistical significance values are available in Supplementary Table S2.

In relation to the primary outcome (the average EMG activity of the affected limbs), statistically significant changes were observed from the baseline data to the data obtained at the end of the treatment in both limbs (Fig. 2). Focusing on the upper limbs, the results showed a significant increase in the average EMG activity of the hemiparetic limbs in the subjects in the sEMG-B group (Fig. 2a), which indicates a higher performance of the extensor muscles of the hand. In contrast, no significant change was observed in the subjects in the control group. Additionally, differences in the treatment effect for the upper limbs were only statistically significant in the hemiparetic limb in comparison to the contralateral (normal) upper limb (*p* < 0.001 and *p* = 0.104, respectively) (Fig. 2b).

**Figure 3:**
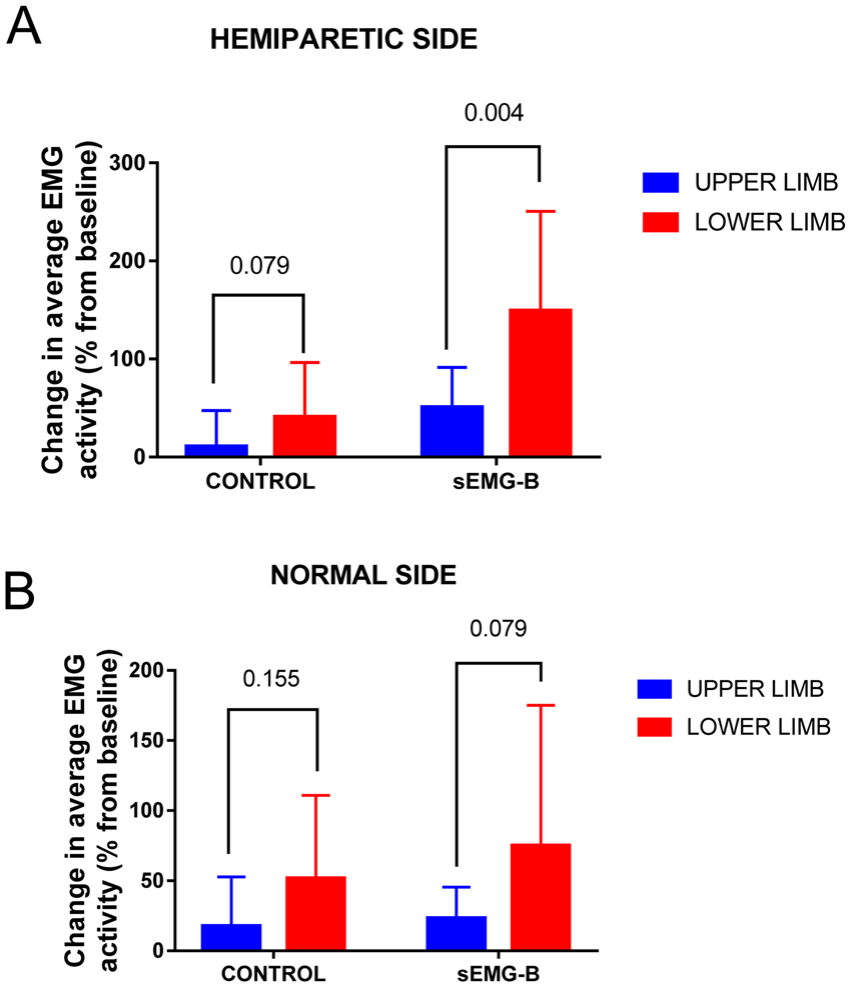
Treatment effect differences between the upper and lower limbs in both Control and sEMG-B groups. Differences were analysed by ANCOVA analysis.

In contrast to the upper limbs, a significant improvement in the EMG activity of the lower limbs was observed in both the control and sEMG-B groups as well as in both the hemiparetic (Fig. 2c) and contralateral (normal) lower limbs (Fig. 2d) (*p* < 0.001). Nevertheless, the differences in the treatment effect were significantly higher in the sEMG-B group. However, the increase in the average EMG activity of the lower limbs was very evident; in fact, a statistically significantly higher increase in EMG activity was observed in the hemiparetic lower limbs in comparison to the upper limbs in the subjects in the sEMG-B group (*p* = 0.004; Fig. 3a). On the contralateral (normal) side, a discrete and non-significant increase in average EMG activity (Fig. 3b) was observed. It is interesting to note that the improvement in muscle activity was not mediated by age, sex or the baseline EMG activity of the subjects, as shown by the ANCOVA analysis; however, the present study’s data revealed an inverse relationship between the baseline Barthel index score and the increase in the muscle activity in the paretic upper limb in the sEMG-B group (*r* = −0.704, *p* = 0.004). This may indicate that the intervention was more effective in the subjects with lower baseline functionality, but also that patients with a lower baseline Barthel score have more room for improvement.

### The efficacy of sEMG-B in the functionality tests

**Figure 4:**
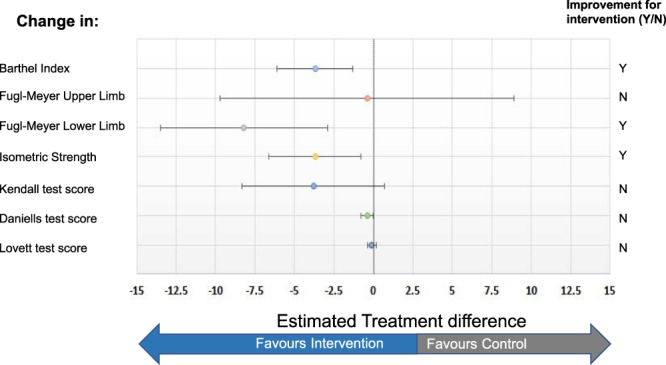
Changes in Barthel index for daily living activity test and the muscle strength functionality tests after 12 weeks of treatment. Forest plot shows estimated treatment differences (ETDs)/odds ratios and 95% CIs. Data are from the full analysis set (completers subjects of control and sEMG-B groups). Data at baseline are mean ± s.d. Improvement/worsening refer to the statistically significant changes from baseline with sEMG-B intervention relative to the control group. Precise data and statistical significance values are available in Supplementary Information Table S3.

In addition to the effect of the intervention on the primary outcome, changes in the functional capacity of the elderly subjects were considered as a secondary outcome. Changes in the Barthel index indicated that the subjects in the sEMG-B group showed a statistically significant increase in their ability to perform basic daily living activities in comparison to the control group (Fig. 4). Moreover, the muscle strength of the wrist was also significantly increased after the intervention. Furthermore, the sEMG-B group exhibited a better performance, as noted in the FM, Daniels and Worthingham’s Muscle Test (DWMT), Kendall Manual Muscle Test (KMMT) and Lovett’s test (LT) scores; however, statistical significance was only observed in the FM scores for the lower limbs.

## Discussion

The present study was conducted to determine the effectiveness of using sEMG-B to increase the muscle activity of the extensor muscles of the hand and the dorsiflexor muscles of the foot in the hemiparetic limbs of elderly patients with brain damage resulting from a stroke. The present study’s data seem to indicate that sEMG-B is a suitable intervention to improve muscle strength, and, therefore, muscle functionality, in elderly patients with brain damage from a stroke.

Biofeedback has been used for more than 50 years in rehabilitation to recover normal movement patterns after injuries^13^. This procedure facilitates the improvement of accuracy during rehabilitation sessions, involves patients in their own rehabilitation tasks and reduces the need to consult the healthcare professional during the programme^10^. Thus, biofeedback can be combined with different physical rehabilitation procedures to improve the efficacy of these methods. One such technique is EMG. While, in conventional EMG, there is an electrical stimulation of the muscle of interest guided by an EMG signal, in biofeedback, a patient can self-identify his/her own muscle activity through the conversion of EMG signals to visual and/or auditory signals. Therefore, patients can control and regulate the muscle activity themselves, which is normally not controllable due to brain damage^12^. Traditionally, sEMG-B has demonstrated its usefulness in improving muscular torque recovery^14^, articular and muscular recovery after surgery^15^, and even in the treatment of pain due to excessive muscular tension^16^.

Similarly, extensive research studies have also focused on investigating the benefits of sEMG-B use in the rehabilitation of hemiplegic patients after a stroke episode. Although some studies have concluded that the use of sEMG-B has no effect on improving functional recovery^10^, other studies have reported promising data. As far back as 1980, Davis and Lee reported a rudimentary biofeedback method to improve flexion-extension movements of the wrist^17^. Recent research, such as the work of Rayegani *et al*.^18^ and the work of Kim^15^, have confirmed the effectiveness of sEMG-biofeedback on upper extremity functions. Other studies also reported on the effectiveness of this intervention in improving the activity of impaired lower limbs after a stroke^19,20^. In fact, the meta-analysis conducted by Stanton *et al*. confirmed, with a high evidence level, greater improvement in lower limb activities with biofeedback therapy^21^.

However, all these previous studies were conducted with adult participants that did not include older patients (>70 years), and, to the best of our knowledge, only the work of Bradley *et al*. has evaluated the effect of EMG-biofeedback on an age-matched population^22^. Moreover, in the work of Bradley *et al*., the intervention was focused on improving gait in post-stroke elderly patients; unfortunately, no significant effect was observed as a consequence of the intervention^22^.

Therefore, the present study is the first to describe a significant improvement in muscle activity in the paretic limbs of post-stroke hemiplegic elderly patients. The lack of studies concerning subjects of advanced age is somewhat surprising if one considers that strokes are more frequent in these subjects^23^; however, it is important to note that, until recently, a stroke was considered to be irremediable and a fatal event in older adults. This fact may be the reason why compassionate attitudes in favour of therapeutic nihilism were imposed in the treatment of elderly patients who experienced a stroke^24^. However, the sEMG-B technique has evolved significantly, and it has several characteristics that are especially relevant for elderly people. First, it is a non-invasive technique, which reduces the risk of side-effects. Second, the instruments and materials are relatively inexpensive, so they can be employed in routine clinical practice to treat these patients.

Restoring extensor and flexor muscle activity is essential for increasing hand and foot functions; however, in elderly people, it is perhaps more important to improve the ability to carry out basic daily living activities, such as personal hygiene, autonomy, etc. Thus, the results from the Barthel index confirmed that although the subjects in both groups improved their ability to perform these activities, the increase was greater in the sEMG-B group, which was likely due to the greater improvement in the upper limbs in comparison to the control group. This fact confirms that the intervention increased the subjects’ muscle activity and their ability to perform basic activities, which, is very important for stroke patients.

These data are in line with the findings reported by Doğan-Aslan *et al*., in which a significantly greater improvement in the Barthel and upper extremity FM scores was demonstrated in the sEMG-B group in comparison to the control group^25^. However, as in previous studies, the average age of the participants in Doğan-Aslan *et al*.’s study was lower than in the present study, which limits the ability to compare the results obtained in both works.

In spite of the growing body of evidence favouring the beneficial effect of sEMG-B therapy, some studies have been unable to detect the significant effect of this therapy in comparison to conventional therapy, such as the case of the meta-analysis conducted by Moreland *et al*.^26^. In that study, the effect of EMG was specifically evaluated in the lower extremities, but no significant effect of EMG was observed on the range of ankle motion, ankle angle during gait, stride length or gait speed.

The present study has several limitations. While, it may seem that the number of subjects included in this study was small, the accuracy of the technique to evaluate muscle activity was very high, so the standard deviation was reduced, limiting the need to enrol more subjects. Moreover, several previous studies employed a similar or even lower number of patients in their work^27–29^. However, in the present study, the estimation of the effect was limited to the outcome that occurred during the time of the intervention, and no follow-up was performed. Undoubtedly, further studies evaluating the long-term effect of sEMG-B on elderly stroke patients would be relevant because the effect of the intervention on other variables of interest could be determined, such as life expectancy and the quality of life of these subjects.

In summary, the present study’s findings suggest that sEMG-B therapy is suitable for improving the muscle activity of the extensor muscles of the hand and the dorsiflexor muscles of the foot in the hemiparetic limbs of post-stroke hemiplegic elderly patients. This increase was reflected in the ability of the study’s participants to perform basic daily living activities. Considering the excellent results obtained, this kind of intervention may be considered to be a potential alternative therapy to be included in efforts to improve the physical conditioning of stroke-derived hemiplegic patients.

## Methods

### Design

This randomised clinical trial was conducted from January–December 2018 at the University Hospital “Sagrado Corazón” of Malaga facilities. Written informed consent was required from each patient to participate in the study. The protocol of this randomised trial adhered to the CONSORT guidelines^30^. The CONSORT checklist is available in the Supplementary Table S1. To evaluate the impact of surface electromyography-biofeedback (sEMG-B), a double-blind (*de facto* masking) trial was designed; neither the participants nor the researcher who carried out the sEMG-B therapy knew the purpose of the study. Participants were unaware of the treatments and possible assignments between the groups. The trial was registered on clinicaltrials.gov (identifier: #NCT03838809. Date of registration: 02/12/2019). A detailed research protocol of the study is available as Supplementary Information.

One researcher (J.J.H.M.) carried out the randomisation. In order to obtain a similar size in both groups, a randomisation in blocks with a 1:1 allocation ratio was performed. For this, 20 sheets with the word CONTROL and 20 sheets with the word INTERVENTION were introduced in envelopes of the same size and colour. The first 40 subjects who were directed to the physiotherapy services of the hospital and consented to take part in the study randomly chose one of the envelopes and gave it to the researcher without opening it. Randomisation divided the participants into two groups, depending on whether they were treated with conventional manual physiotherapy techniques (control group) or followed an intervention based on the sEMG-B technique (sEMG-B group). The day before the beginning of the intervention, the participants performed a series of mobility and functionality tests, as described below. After 12 weeks of intervention, all participants were re-examined to measure their performance on the mobility tests and to evaluate possible changes in the functionality parameters.

### Participants

The sample size required for the study was determined with the help of the GPower 3.0 program^31^. The sample size was estimated using a two-sided *F-*test with a significance level of 95%, considering a statistical power (β) of 80% and a between-group treatment effect difference (d) of 10 µV average EMG activity. A standard deviation (σ) of 10 µV was assumed, following a previous work conducted with the same patients^19^. This procedure designated a total of 12 subjects per group. Figure 1 shows the flow diagram for the selection of the subjects of this study. Finally, 40 subjects derived from the Neurological Service of the hospital took part in the study.

Selection criteria included patients with ischaemic stroke confirmed by computed tomography or magnetic resonance imaging, engaged to physiotherapy services between two and six weeks after the stroke (mean time after stroke 20 ± 2 days), between 75–85 years-of-age, diagnosed of acquired brain damage as a consequence of a stroke, and having significant limitation in the extension of the hand and in the dorsiflexion of the foot as a consequence of the stroke without spasticity (Ashworth scale 1 or 1+). Voluntarily participation in the study was also considered as a selection criterion. The exclusion criteria included patients with haemorrhagic stroke, an undetectable surface EMG signal (<0.5 µV), a previous history of neurologic comorbidity that might impair muscle strength (lateral amyotrophic sclerosis, muscular dystrophy, myasthenia gravis, and spinal muscular atrophy) and taking any medication known to affect muscle strength (muscle relaxants, antiseizures, antispasticity, anxiolytics, or antihistamines). Pacemaker patients were also excluded from the study. Finally, those patients with severe cognitive decline or dementia or other psychiatric conditions and patients with severe visual and/or hearing impairment (beyond the aging-related deterioration) were excluded. The evaluation of cognitive and sensory states was performed in the same Neurological Service. All subjects were right-handed and the hemiparetic side was the left-side. None had previous experience in isometric evaluations.

The present work was carried out with previous written authorisation from the Catholic University of Murcia’s Ethics Committee (available as Supplementary Information). All research was performed in accordance with local legislation on biomedical research (Spanish Law 14/2007 about Biomedical Research). Patients were informed about the design of the study orally and in written form. Consent to participate in the study was also requested in both ways. An explanation of the research project in the ethical sense was also given, informing them about the aim of the results obtained, warranting confidentiality and anonymity of the data, and respecting the Helsinki Declaration Agreement.

### Outcome measures

The primary efficacy end-point was a change in the functionality of the upper and lower limbs, measured as changes in the mean activity of the EMG signals. This outcome represents the overall average microvolts achieved during the all work periods of a sEMG-B session. This was selected as the main outcome because, generally, the higher the work average is, the better the muscle performance. *A priori* secondary efficacy end-points included changes in the Barthel index scores and in the other muscle function tests from the baseline to the end of the treatment.

### Intervention

All of the patients from both the sEMG-B and control groups were initially evaluated to determine their average muscle activity or strength by electromyography. The patients were monitored at the baseline and at the end of treatment. Control and sEMG-B groups followed an intervention based on isokinetic exercises with an elastic band and stretching exercises.

In the control group, the sessions were organized to perform rehabilitation exercises on the hand for 30 minutes and on the foot for another 30 minutes, for a total of 1 hour per session. In the sEMG-B group, physiotherapy rehabilitation sessions lasted 15 minutes for the hand and 15 minutes for the foot. Subsequently, the intervention of sEMG-B was performed in similar sessions of 15 minutes for the hemiplegic hand and foot, for a total of 1 hour per session.

The rehabilitation exercises of the hand included exercises with elastic band to strengthen extensor muscles of the fingers and wrist, self-assisted active mobilization exercises, self-assisted and active wrist extension exercises performed with a spiked surface sensory ball for soft stimulation. Subsequently, stretches of the flexor muscles were performed and the wrist was mobilized passively, in a way that helped us to induce an improvement in the wrist extension. Besides, the exercises performed on the hemiparetic foot included strengthening exercises for the flexor muscles of the foot with elastic band, self-assisted active exercises for ankle flexion with sensory ball with spiked surface for soft stimulation performed with a spiked surface sensory ball for soft stimulation and stretching exercises on the extensor muscles of the foot. Finally, passive stretching of the extensor muscles of the foot was carried out to us improve the flexion movement of the hemiparetic foot.

For the EMG evaluation and intervention, the Neurotrans Myoplus 2 Pro System (Verity Medical Ltd, UK) was employed. The intervention in the sEMG-B group was conducted in a 15-minute session for the upper limbs and an additional 15-minute session for the lower limbs with a conventional sEMG programme, plus an auto-induced (biofeedback) stimulation EMG. Each session was divided into periods of five seconds of relaxation followed by 15 seconds of activity. At the beginning of the session, the subjects were asked to contract the muscle with as much strength as possible for three seconds, which was considered to be the maximum voluntary isometric contraction (MVIC). This MVIC value was established as the threshold value for data normalisation, since this is the most reliable method to determine the differences in muscle activation, especially when testing over multiple sessions^32^. The muscle activity of the subjects was monitored in a screen as visual and auditory signals. When the muscle activity was below the activity threshold, an alarm cautioned the subject to increase his/her muscle activity. Considering the advanced age of the subjects, verbal feedback was provided by the therapist, in order to avoid possible hearing issues derived from the subjects’ age. The sessions were conducted twice a week over a period of three months (24 sessions in total). For the control group, the intervention consisted of a similar duration of time and number of sessions.

The subjects were instructed to carry out the sEMG activity. A chair with a backrest and a side table were used. The subject remained in a seated position with 90° flexion of the knees and hips while the upper limbs held 90° flexion of the elbows and forearms in pronation. The electrodes were placed 2 cm towards the caudal direction of the external epicondyle of the elbow and on the anterior area of the wrist, between the styloid process of the ulna and the styloid process of the radius. For the lower limbs, the electrodes were placed 2 cm towards the caudal direction of the external tibia tuberosity and on the anterior area of the ankle at an intermediate point between the external and internal malleolus. The positive electrode was placed in the distal area, and the negative electrode was placed in the proximal zone of both the upper limbs and the lower limbs.

### Measures

The mean EMG activity of the extensor and dorsiflexor muscles during all activity stages of each EMG session was analysed with the same instrument (Neurotrans Myoplus 2 Pro System, Verity Medical Ltd, UK) that was used for the intervention, which was considered as an indicator of average muscle activity. The calculation excluded the first second of each activity period to eliminate the deviation from the first contraction attempt. The accuracy of the EMG signals was 0.1 µV. The isometric strength (Nw) of the hand was assessed with a hand-held digital dynamometer (Smedley digital hand dynamometer, RMS Ltd., UK). The measurement was performed three times consecutively, with a 2–3-minute interval between measurements. Peak force values were recorded for each trial, and the median value was considered as the standard value, following the manufacturer’s instructions.

The evaluation of the physical performance was conducted using the FM evaluation test^33^. The Fugl-Meyer Assessment for the Upper Extremity (FMA-UE) and the Lower Extremity (FMA-LE) tests were employed^34^. The stroke patients with better physical performance had higher scores. In addition to the FMA tests, which are specifically designed for post-stroke hemiplegic patients, several scales that are used to evaluate muscle strength in patients with brain damage were employed. Specifically, the DWMT^35^, the LT^36^ and the KMMT^37^ were conducted. The DWMT and LT are scored ranging from 0–5, indicating the total absence of muscle contraction (score = 0) to the highest muscular contraction (score = 5). The KMMT is scored in percentages with similar criteria, ranging from the absence of muscle contraction (score = 0%) to the highest level of contraction (score = 100%). These scales provide a general assessment of muscle strength. They do not specifically measure the strength of a single muscle; instead, the muscle strength is measured through joint movement. Lower scores (0 or 0%) may indicate the absence of contraction, while higher scores (5 or 100%) indicate normal strength with active movement of the joint that overcomes gravity and maximum resistance.

Finally, the ability of the patients to perform basic daily living activities was determined through the Barthel index, which, in its Spanish translation, provided a Cronbach’s alpha greater than 0.70^38^.

### Statistical analysis

Considering the reduced final sample size, normality was assessed through the Shapiro-Wilk test, which revealed that the primary outcome variable was normally distributed. Therefore, parametric tests were employed. First, a basic descriptive statistical analysis was performed to evaluate the general characteristics of the study population. Possible baseline characteristic differences between both groups were analysed by means of a Student’s *t-*test. The efficacy analyses were performed on the data from the completers set, which included all participants that underwent the entirety of the intervention sessions. The present results are based on this analysis set unless otherwise noted. The same *t*-test was used to determine possible differences in the change of mobility and functionality parameters at the end of the treatment compared to the baseline values (Δ parameter = final value - baseline value) to assess possible estimated treatment effect differences between the groups.

To evaluate the mean limb activity changes, an *a priori* two-way (time x group) ANCOVA analysis was carried out in order to determine a possible interaction between the activity changes and the treatment groups. This test was also conducted to exclude possible bias due to age, sex, and other clinical antecedents. Baseline characteristics were further considered as covariates. All statistical tests were two-tailed and were performed considering a significance level of *p* < 0.05. The analysis was carried out with the help of the SPSS software for statistical analysis (version 24.0.7, SPSS Inc., Chicago, IL).

### Clinical trial information

The trial was registered on clinicaltrials.gov (Registration Number: #NCT03838809. Registration date: 02/12/2019). More information is available at: https://clinicaltrials.gov/ct2/show/NCT03838809.

## Supplementary information

CONSORT checklist and detailed statistical data

## Data Availability

The datasets generated and/or analysed during the current study are available from the corresponding author upon reasonable request.
